# The Importance of Food for Endotoxemia and an Inflammatory Response

**DOI:** 10.3390/ijms22179562

**Published:** 2021-09-03

**Authors:** Charlotte Erlanson-Albertsson, Karin G. Stenkula

**Affiliations:** 1 Appetite Control, Department of Experimental Medical Science, BMC, Lund University, 221 84 Lund, Sweden; 2 Glucose Transport and Protein Trafficking, Department of Experimental Medical Science, BMC, Lund University, 221 84 Lund, Sweden; karin.stenkula@med.lu.se

**Keywords:** obesity, virus, saturated fat, plant-based diet, meat, Western diet

## Abstract

Bacterial endotoxin is a potent inflammatory antigen abundant in the human intestine. Endotoxins circulate in the blood at low concentrations in all healthy individuals. Elevated levels of circulatory endotoxins may cause inflammation with the development of chronic disease, either affecting metabolism, neurological disease, or resistance to viral and bacterial infections. The most important endotoxin is LPS, being a superantigen. In this narrative review, the effect of various food components to postprandially elevate circulating LPS and inflammatory markers is described. There is evidence that the intake of food enriched in fat, in particular saturated fat, may elevate LPS and pro-inflammatory markers. This occurs in both normal-weight and obese subjects. In obese subjects, inflammatory markers are already elevated before meal consumption. The importance of food choice for endotoxemia and inflammatory response is discussed.

## 1. Introduction

Endotoxemia and hyperinflammation are important factors for determining the severity of infectious and non-infectious disease [1,2]. The most important endotoxin is LPS, lipopolysaccharide, which is present at high concentrations in the intestine as part of the intestinal bacterial flora. The LPS from these bacteria can induce a chronic subclinical inflammatory process under certain circumstances [2]. Normally, LPS stays within the intestinal lumen, but can reach the circulation in high concentrations following disruption of the intestinal barrier. This occurs during inflammatory diseases, like allergy and autoimmunity [3], and during chronic inflammatory bowel disease [4], the severity of the disease being correlated to the levels of endotoxemia and inflammatory markers. During type-1 diabetes and chronic fatigue syndrome, a disrupted intestinal barrier is likewise considered a source for the systemic immune activation characteristic for these diseases [5]. An altered intestinal bacterial flora, named dysbiosis, could modify the intestinal mucosa to become thinner and more permeable to pathogens with the establishment of low-grade inflammation [6]. 

In this review, we have focused on the direct role of nutrients to induce endotoxemia and/or elevate the blood levels of pro-inflammatory markers. To this end, meal studies have been collected and carefully reviewed so as to understand more in detail if any such effect is at hand. 

## 2. Aim

The aim of the following review is to describe studies that have investigated the link between food intake and endotoxemia/inflammation. 

## 3. Nutrients and Endotoxemia

### 3.1. The Superantigen LPS

The most common endotoxin is LPS (lipopolysaccharide). Chemically, LPS is a glycolipid located in the outer membrane of the bacterial cell wall in Gram-negative bacteria. LPS reacts with Toll-like receptors (TLR4), whereupon an immunological reaction starts [7,8]. It has been estimated that there is 1 g of LPS in the intestinal lumen in humans [8]. Under normal circumstances, low amounts of LPS are transferred to the circulatory system without causing any inflammatory reaction. During certain conditions, the transfer of LPS from the intestine to the blood is elevated. The diet may assist in this transfer, and it has been described that high-fat diets may contribute to endotoxemia caused by elevated levels of LPS [8]. During metabolic disease like diabetes and obesity, LPS has been identified as a triggering factor for the inflammation [9]. In a recent study, LPS was found to bind to the spike protein S from SARS-CoV-2 virus [10]. Upon binding, large aggregates were formed, consisting of the spike protein from SARS-CoV-2 and several molecules LPS. The large complex gives a strong immunological reaction [10]. Thus, a hyper-inflammatory condition may ensue following the activation of LPS. 

### 3.2. Endotoxemia and Feeding

To investigate the importance of nutrients for the uptake of endotoxins, healthy individuals were served various meals, whereafter endotoxins and inflammation markers were measured in blood. It appears that the composition of meals influences the uptake of endotoxins to the blood, where dietary fat promotes the uptake of endotoxins, and dietary fibers restrict this uptake [11,12,13,14,15]. The results are illustrated through the detailed description below.

### 3.3. Endotoxemia after a Standard Breakfast

In one study, 12 healthy men, 25–30 years in age, received a breakfast at 9.00 o’clock in the morning. The breakfast contained 880 kcal, assembled in a 200 mL nutrition formula, containing 33 g of fat (23 g margarine, 9 g butter, and 1 g olive oil). This was eaten with 75 g of bread, 20 g of jam, and 200 g of banana [11]. Blood sampling demonstrated the level of endotoxins to increase postprandially, being two-fold higher after one hour. Simultaneously, an inflammatory response was revealed through the elevation of interleukin-6 (IL-6), a pro-inflammatory hormone. The elevation of the inflammatory marker started after two hours and ended after four hours. Electron microscopy demonstrated LPS was present in the chylomicrons carrying the dietary fat, being absorbed from the intestine for distribution into the body. Cellular studies established that the uptake of LPS into the intestinal cells was higher with highly dispersed fat [11]. The authors drew the conclusion that a standard breakfast with bread, butter, and sweet jam causes LPS elevation and an inflammatory response.

In another study, twelve healthy normal weight participants (mean BMI of 23 kg/m^2^ and a mean age of 32 years) were offered a breakfast containing 900 kcal, consisting of a cup of tea and three slices of toast spread with a total of 50 g butter. Measurement of LPS in the circulating blood demonstrated a significant rise from low basic levels of around 8 ng/L to approximately 12 ng/L postprandially within four hours after starting the meal. Smoking during the breakfast did not influence the LPS elevation following the breakfast [13]. The authors drew the conclusion that a standard breakfast meal containing butter and bread causes endotoxemia. 

In a third study, 48 healthy normal-weight men of age 25–47 years were, after over-night fasting, offered to consume a drink of 300 kcal containing either cream, glucose juice, orange juice, or water (0 kcal). They had ten minutes to swallow the drink. Thereafter, the levels of endotoxins were determined. It was found that with cream, the LPS level was increased 40% during five hours postprandially, while the glucose juice, the orange juice, or water had no influence [12]. Cream consists of 70% saturated fat, 28% unsaturated fat, and <2% protein. The authors concluded that cream is an essential nutrient to raise endotoxin levels postprandially.

### 3.4. Endotoxemia after a Breakfast Containing Fibers

In a cross-over study, the influence of dietary fiber on the levels of endotoxins was investigated [14]. Healthy normal-weight (BMI < 25) men, 25–50 years in age, were offered two different meals (both with 910 kcal); one a standard American meal and another a fiber-enriched meal recommended by the American Heart Association (AHA) [14]. The standard meal contained two breakfast muffins, egg, sausage, and hash browns, 41% carbohydrate, 42% fat, and 17% protein. The fiber-rich meal consisted of oatmeal, milk, raisin, muffins with peanut butter, and orange juice, 58% carbohydrate, 27% fat, and 15% protein. During the meal, blood was sampled for the determination of metabolic and inflammatory parameters. Blood glucose values were higher after consumption of the standard meal compared to the AHA-meal, despite the lower level of carbohydrate consumed. This is explained by the absence of fiber in the standard meal, in contrast to the AHA-meal, which contained fiber from both raisin and from oat [14]. Insulin and cholesterol levels were the same during the two different meals. Comparatively, the triglyceride serum levels were significantly higher in the standard meal compared to the AHA-meal, measured three hours after the start of the meal. The basal endotoxin levels in these men were around 0.35 EU/mL and increased by 40% after the standard meal, and with 10% after the AHA-meal [14]. Based on these findings, it was concluded that fiber included in a breakfast may restrict the elevation of endotoxin levels in the blood compared to a standard breakfast.

Together, these studies demonstrate the ability of a breakfast containing bread and butter to raise endotoxin levels postprandially and induce an inflammatory response in normal weight men. The question is if any specific nutrient is important for the uptake of LPS from the intestine, and if this could be modified.

### 3.5. The Mechanism of Postprandial Uptake of LPS

**Figure 1:**
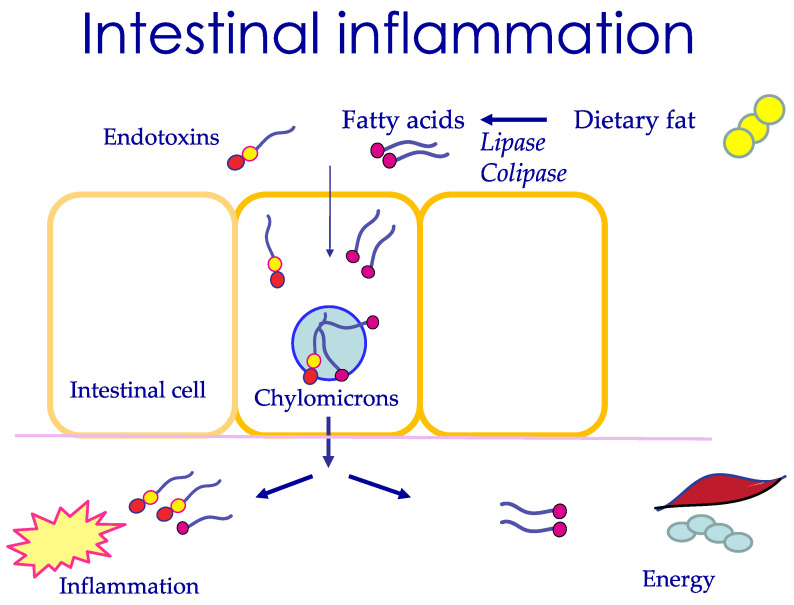
Intestinal uptake of endotoxins occurs with uptake of dietary fat after this has been hydrolyzed by pancreatic lipase and its protein cofactor lipase to fatty acids. In the intestinal cell, chylomicrons are formed and leave through passage into the lymph. Endotoxins have the potential to induce inflammation. Fatty acids are primarily used as an energy source but may also induce inflammation.

There may be several pathways for elevation of endotoxin levels in the blood following the consumption of a standard Western breakfast of bread, butter, and milk [11,13]. Chemically, LPS resembles a glycolipid, and the absorption of LPS follows other dietary lipids in the physiological process of intestinal lipid uptake. This occurs through the digestion of dietary lipids by lipase enzymes, mainly by pancreatic lipase and its co-factor colipase, followed by uptake and reconstitution of the lipids into large particles, chylomicrons, released into the lymph for distribution in the body. LPS has been identified in chylomicrons, suggesting a trans-cellular uptake of LPS [15,16], as illustrated in a simplified model, as shown in Figure 1. The uptake of LPS through chylomicrons is a slow process, occurring over 3–4 h. The chylomicrons are transported to the lymph and from there are cleared by the liver. The LPS is protected as long as it stays in the chylomicrons, but once released in the circulating blood, it may start an inflammatory action. The chylomicrons are disintegrated through the action of lipoprotein lipase situated in the blood vessel wall, a degradation that may occur along the way to the liver.

In addition to an uptake via chylomicrons, it appears that LPS is taken up directly to the intestinal cell and released into the blood [17]. This trans-cellular uptake of LPS is rapid and occurs within 30 min. The uptake requires long-chain fatty acids and bile salt in the intestinal lumen, thus being linked to the consumption of fat [17]. In contrast to chylomicrons, LPS through this molecular uptake is however ready to react and start an inflammatory process. Therefore, factors that limit this trans-cellular uptake are important. It has been demonstrated that the intestinal hormone GLP-2, released from L-cells, limits the trans-cellular uptake of LPS in vitro [17]. There may also be a para-cellular uptake of lipids, demonstrated through a change in tight junctions, established in an in vitro model [18]. 

It is concluded that LPS is taken up into the circulation during normal lipid absorption, likely through a trans-cellular slow pathway forming chylomicrons or a trans-cellular rapid molecular uptake (Figure 1). While chylomicrons are protective of the action of LPS, the trans-cellular and para-cellular molecular uptake of LPS transmit risks for an inflammatory response of the body. The necessity of a dietary fat component for LPS uptake in healthy individuals is supported by studies where LPS appeared in the blood following the consumption of cream, but not glucose juice orange juice [12].

### 3.6. Nutrients That Protect the Uptake of LPS

**Figure 2:**
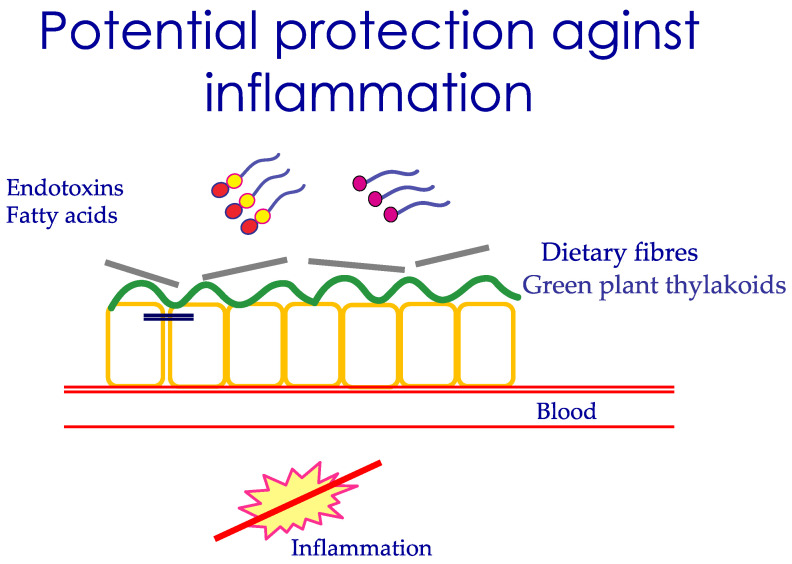
Schematic picture to demonstrate the ability of dietary fibers and green plant thylakoids to adhere to the intestinal mucosa, reducing the rate of uptake of fatty acids and potentially the uptake of endotoxins, leading to reduced inflammation.

A relevant question is whether any nutrients or hormones could protect the uptake of LPS, in particular the molecular uptake of LPS. It has been demonstrated that dietary fiber delays both fat digestion and fat absorption [19], crucial events to restrict the uptake of LPS. Further, thylakoids extracted from green leaves in a similar way delay fat digestion and form a distinct layer on the intestinal mucosa, restricting the uptake of nutrient molecules [20,21]. This covering effect of fibers and thylakoids potentially restricts the uptake of LPS and of fatty acids by the intestinal cell, as schematically drawn in Figure 2. Indeed, the addition of fiber in the AHA recommended meal restricted the uptake of LPS [13].

### 3.7. Endotoxemia Postprandially during Obesity

Since obesity has been described as a low-inflammatory state, the influence of a fat-rich meal on endotoxemia and inflammation was investigated in obese subjects and compared to normal-weight subjects [15]. A breakfast was served to normal-weight men (*n* = 8) and to obese men (*n* = 8) and the levels of endotoxins measured postprandially [15]. The breakfast consisted of bread, either 10 g or 40 g butter, and skim milk. The breakfast with 40 g butter contained 215 kcal, the energy distributed as 67% fat, 26% carbohydrate, and 7% protein. Five hours later, a lunch was served containing pasta, turkey, olive oil, bread, butter, and fruit salad containing 713 kcal energy distributed as 29% fat, 51% carbohydrate, and 20% protein. The meals were eaten over ten minutes. 

The uptake of fat from the intestine and the levels of LPS and markers of inflammation were measured, both after breakfast and after lunch. The normal-weight men had a certain uptake of LPS in the blood independent of the uptake of dietary fat. The obese men demonstrated an uptake of LPS into the blood related to the amount of fat taken up from the intestine. The LPS concentrations in the blood remained elevated during the observation period of eight hours. The LPS levels were higher in obese men compared to normal-weight men, and were enriched in chylomicrons secreted from the intestine. The uptake of LPS therefore occurred trans-cellularly and not via any para-cellular transport, verified by measurement of zonulin, a molecule used for measuring intestinal permeability [15]. In another study, LPS levels were measured in both serum and the chylomicron fraction at baseline and 3 h after a fat overload meal containing 50 g fat in morbidly obese subjects [22]. Postprandially, serum and chylomicron LPS increased, and this increase positively correlated to postprandial triglyceride levels. These results support chylomicron-mediated transport of LPS, since subjects with the highest fold increase in triglyceride levels had higher levels of postprandial chylomicron LPS.

### 3.8. Endotoxemia and Postprandial Inflammation, a Tipping Point in the Obese State?

Obesity is a state of low-grade inflammation, where overconsumption of food initially triggers the adipose tissue to increase the size of fat cells in a healthy way [23]. However, continued overconsumption leads to an accumulation of very large fat cells and an increased release of inflammatory cytokines from both the enlarged fat cells and from the inflammatory cells residing in the adipose tissue [23]. In particular, visceral adipose tissue produces multiple pro-inflammatory cytokines, like TNFα, IL-6, and IL-8 [24]. Increased circulating levels of LPS in obese individuals could contribute to the inflammatory response in fat tissue via binding of LPS to TLR4 present in macrophages and fat cells [25]. Furthermore, increased concentrations of serum LBP, an LPS-binding protein (LBP), which forms a complex with LPS to enhance binding to CD14 and TLR4, were found in overweight and obese subjects [26].

Possible meal-induced inflammation has been investigated in connection with established endotoxemia. It was found that the pro-inflammatory hormone IL-6 was elevated postprandially in normal-weight men [11,15]. Yet, there was no inflammatory response in obese men postprandially. Instead, the basal levels of IL-6 were elevated compared to normal-weight men [15]. A low-grade, chronic inflammation in the obese condition could lead to a defect immune system. For example, increased levels of IL-6 and leptin contribute to impaired function and altered phenotype of the so-called natural killer (NK) cells, key players in the innate immune system fighting against bacteria and virus-infected cells [27]. 

### 3.9. Long-Chain Fatty Acids May Be the Trigger for Inflammation

The role of LPS as a major trigger for post-prandial inflammation has been questioned [28]. In a study of 62 normal-weight volunteers, a typical American meal was given with 36% kcal fat, whereafter LPS, fatty acids, and inflammatory markers were measured in plasma. It was found that inflammatory markers like IL-6, IL-8, and TNFα were all elevated postprandially, as was the level of free fatty acids, whereas no change in the levels of endotoxins was observed. Further analysis indicated that the major fatty acid giving postprandial inflammation was C16:0, palmitic acid, a saturated fatty acid present in animal fat and various food additives to obtain creaminess [28]. Nutrients containing saturated fat hence may be the major trigger for post-prandial inflammation, illustrated in Figure 1. 

### 3.10. Nutrients, Intestinal Permeability, Microbiome, and LPS

Nutrients affect the intestinal barrier function in a long-term manner, not only during single meals. This occurs through their influence on the microbiota composition, which may either be healthy or more damaging for the intestine. The role of diet in affecting microbiota versus other factors has been estimated to be around 60%. The colon contains most bacteria, and the normal composition is *Firmicutes*, 64%, *Bacteroidetes*, 23%, followed by *Actinobacteria* and *Proteobacteria* [6]. A high amount of long-chain fatty acids, in particular saturated fatty acids in the intestine, results in reduced microbial diversity, and an increased abundance of Gram-negative bacteria, like *Firmicutes* [29]. With these bacteria, there is a large production of LPS, which could be absorbed by the presence of dietary fat in the intestine [30]. Not all fat is equally inflammatory, for example, olive oil is less harmful than butter [31]. The role of a high-fat diet in dysbiosis and disruption of the intestinal barrier has been documented earlier [32]. Eastern diets based on carbohydrates derived from plants, vegetables, rice, and fruits shape another microbiota. Eastern populations have a higher prevalence of *Prevotella* than *Bacteroides* compared to the Western population [30]. Simple carbohydrates and fiber are mostly associated with an increase of *Prevotella*, suggested as a health-promoting bacteria [33]. In contrast, diets with animal sources of protein and fat are associated with an increased number of *Bacteroides*, which are more prone to disease [30]. A diet rich in vegetables and fibers reduces intestinal pH and protects against the growth of pathogenic bacteria. Microbiota is therefore closely coupled to food quality by determining the luminal antigens exposed to the intestine. The impact of diet on the severity of an infectious disease has been described in a six-country study, where individuals following a plant-based diet with higher intake of vegetables, legumes, and nuts, and low intake of animal products, were reported to have a lower risk of developing moderate and severe disease compared to individuals that did not follow these diets [34]. A suggested mechanism is the ability of plant-rich food to cover the intestinal mucosa, hence restricting the inflammation induced by dietary fat and/or endotoxins, as illustrated in Figure 2.

## 4. Conclusions

In conclusion, food consumption may be important for endotoxemia and low-grade inflammation. This may occur through the meals eaten, and/or through the microbiota established. Diets with large amounts of saturated fat, animal products, and refined carbohydrate may induce endotoxemia more markedly than diets containing fiber-rich plant-based food. Whether this latter type of food pattern is relevant for suppressing inflammation during infectious disease is not known and awaits further studies.
